# Pasireotide treatment significantly reduces tumor volume in patients with Cushing’s disease: results from a Phase 3 study

**DOI:** 10.1007/s11102-019-01021-2

**Published:** 2019-12-24

**Authors:** André Lacroix, Feng Gu, Jochen Schopohl, Albert Kandra, Alberto M. Pedroncelli, Lixian Jin, Rosario Pivonello

**Affiliations:** 1 Division of Endocrinology, Department of Medicine, Centre hospitalier de l’Université de Montréal (CHUM), 900, rue Saint-Denis, Room R08-474, Montréal, QC H2X 0A9 Canada; 2 Department of Endocrinology, Key Laboratory of Endocrinology, Ministry of Health, Peking Union Medical College Hospital, Beijing, China; 3 Medizinsche Klinik IV, Ludwig‐Maximilians Universität München, Munich, Germany; 4 Novartis Pharma AG, Basel, Switzerland; 5 Novartis Pharmaceuticals Corporation, East Hanover, NJ USA; 6 Dipartimento Di Medicina Clinica E Chirurgia, Sezione Di Endocrinologia, Università Federico II Di Napoli, Naples, Italy

**Keywords:** Cushing’s disease, Pasireotide, Corticotroph tumor volume, Urinary-free cortisol

## Abstract

**Purpose:**

In the multinational, randomized, double-blind, Phase 3 B2305 study of patients with Cushing’s disease (CD; ClinicalTrials.gov identifier NCT00434148), pasireotide substantially decreased urinary-free cortisol (UFC) levels, decreased mean corticotroph tumor volume, and improved clinical signs of disease. The current post hoc analysis further assesses the effects of pasireotide on corticotroph pituitary tumor volume.

**Methods:**

Patients enrolled in the B2305 study had persistent or recurrent CD or newly diagnosed CD but were not surgical candidates. Enrollees were randomized to receive subcutaneous pasireotide, either 600-μg or 900-μg twice daily. Tumor volume was assessed independently at months 6 and 12 by 2 blinded radiologists and compared with baseline value and UFC response.

**Results:**

Of 162 patients enrolled in the trial, 53 had measurable tumor volume data and were included in the post hoc analysis. Reductions in tumor volume were both dose and time dependent. Tumor volume reduction was more frequently observed at month 6 in the 900-μg group (75%) than in the 600-μg group (44%). Similarly, at month 12 (n = 32), tumor volume reduction was observed more frequently in the 900-µg group (89%) than in the 600-µg group (50%). Control of UFC levels was not required for reduction of tumor volume. No relationship was noted between baseline tumor size and change in tumor size.

**Conclusions:**

Measurable decreases in pituitary tumor volume were observed in a large proportion of patients with CD and measurable tumor volume who were enrolled in the trial and treated with subcutaneous pasireotide; this decrease was not correlated with UFC control.

**ClinicalTrials.gov identifier:**

NCT00434148.

## Introduction

Cushing’s disease (CD), the most frequent cause of endogenous Cushing’s syndrome, is characterized by excess production of adrenocorticotropic hormone (ACTH) by a corticotroph pituitary tumor, leading to hypersecretion of cortisol by the adrenal glands [1–6]. CD is associated with a high burden of comorbidities, and uncontrolled CD is associated with increased morbidity and approximately a two to fivefold greater risk of death compared with controlled populations [1, 3, 5–8].

Selective transsphenoidal surgical removal of the corticotroph tumor is the recommended initial treatment for patients with CD [2, 3, 5, 6, 9, 10]. Optimal surgical results depend on the expertise of the surgeon, with remission rates of 48.7% to 100% (mean 82.1%; median 85.7%) observed in patients with pituitary microadenomas and 30.8% to 100% (mean 62.3%; median 64.1%) in patients with macroadenomas [6]. Recurrence rates of 0% to 36.4% (mean 11.7%; median 10.9%) and 0% to 59% (mean 18.8%; median 13.9%) have been reported following initial remission in patients with microadenomas and macroadenomas, respectively. Long-term surgical failure, which includes disease persistence and recurrence after surgery, is reported to be greater in patients with macroadenomas (range 0–71.4%; mean 48.8%; median 52.2%) than in those with microadenomas (range 0–55.5%; mean 25%; median 21.2%). Repeat surgery is an option in the treatment of patients with persistent/recurrent disease, but it is associated with only 58% mean remission rates over an average of 5 years of follow-up and higher rates of complications, including pituitary insufficiency in an average of 38% of patients [2, 5, 6].

Because of their continued exposure to elevated cortisol levels, patients with persistent or recurrent CD pose a significant challenge for management. Optimal care depends on the effective use of other available therapeutic options, including repeat pituitary surgery, radiotherapy, or bilateral adrenalectomy, all of which should be adapted to each patient’s specific situation [2, 5, 6, 9–12].

Medical therapy is also an option for treatment of patients with CD; to date, the largest body of clinical experience has been obtained with the steroidogenesis inhibitors metyrapone and ketoconazole [2, 13–15]. In a limited number of retrospective studies in patients with CD, mean response rates were 71% for metyrapone and 64% for ketoconazole [6]. However, disadvantages of adrenal-directed medical therapy are that it does not treat the underlying tumor that causes CD and is associated with various adverse events including gastrointestinal-related events, hypokalemia, hypogonadism, hirsutism, and elevations in liver function test results [2, 10]. It has been proposed that pituitary-directed medical therapies may provide broader clinical benefit including tumor control as well as reversal of hypercortisolism [2, 13, 16].

Somatostatin receptors (SSRs) are highly expressed in corticotroph pituitary tumors—particularly SSR type 5 (SSR_5_)—and are therefore a logical target for pharmacologic inhibition [2, 4, 10, 16]. Pasireotide is a multireceptor-targeted somatostatin analog (SSA) that binds with high affinity to 4 of the 5 types of human SSR, but exhibits the greatest binding affinity for SSR_5_ [2, 10, 16, 17]. In corticotroph tumors, pasireotide binds to and activates the SSRs, resulting in inhibition of ACTH secretion, which in turn leads to decreased cortisol secretion [17]. In a Phase 3 trial in patients with CD (B2305; ClinicalTrials.gov identifier NCT00434148), a 6-month treatment with subcutaneous (SC) pasireotide was associated with normalization of urinary-free cortisol (UFC) levels without the need for dose adjustment in 15% of patients treated with a 600-μg twice-daily (BID) dose and in 26% of patients treated with a 900-μg BID dose [18]. Marked improvements in signs and symptoms of CD as well as reductions in pituitary tumor volume were also observed in patients enrolled in the clinical trial. Further analysis of data from the trial demonstrated that significant improvements in signs and symptoms of CD were associated with progressive changes in UFC levels following pasireotide treatment [19]. The current report describes the results of a post hoc analysis that evaluated the effects of pasireotide on tumor volume by baseline tumor size in patients enrolled in the trial who had tumors at baseline that were measurable by magnetic resonance imaging (MRI). In addition, the potential association between pasireotide-induced tumor volume control and UFC control was analyzed.

## Methods

**Fig. 1:**
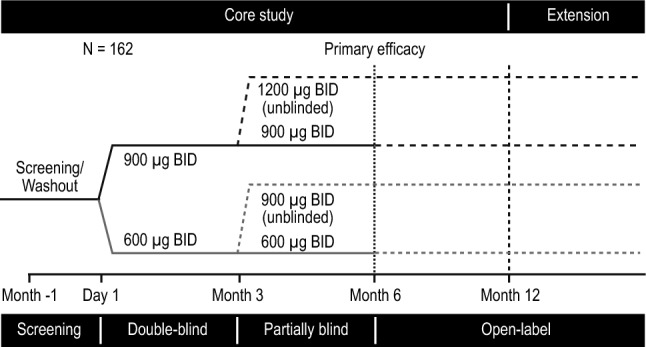
Study design of the Phase 3 study. *BID* twice daily

The B2305 study was a multinational, randomized, double-blind trial evaluating control of UFC by pasireotide in patients with confirmed persistent or recurrent CD or those with newly diagnosed CD who were not candidates for surgery (Fig. 1; full description of the trial design has been published previously) [18]. Patients were eligible for inclusion in the study if they were ≥ 18 years of age and had confirmed persistent or recurrent CD after surgery or newly diagnosed CD if they were not candidates for surgery. Inclusion criteria for active CD included a mean 24-h UFC level ≥ 1.5 times the upper limit of the normal (ULN) range, which was determined from four 24-h samples collected within 2 weeks; a morning plasma corticotropin level ≥ 5 ng/L (1.1 nmol/L); and a pituitary source of Cushing’s syndrome, which was confirmed through MRI identification of a pituitary macroadenoma, by bilateral inferior petrosal sinus sampling central-to-peripheral ACTH gradient > 2 basally or > 3 after corticotropin-releasing hormone or desmopressin stimulation in patients with microadenomas, or by histopathologic confirmation of an ACTH-staining tumor [18].

Key exclusion criteria were pituitary irradiation within the previous 10 years, compression of the optic chiasm with secondary visual field defects, symptomatic cholelithiasis, and glycated hemoglobin level > 8% [18]. Patients who were receiving medical treatment for CD must have completed a washout period prior to baseline efficacy assessment [18]. The study was approved by independent review committees at the participating institutions and complied with the Declaration of Helsinki, the Harmonized Tripartite Guidelines for Good Clinical Practice from the International Conference on Harmonization, and local laws. Each patient provided written informed consent.

Patients were randomly assigned to receive BID SC injections of pasireotide at doses of either 600 μg or 900 μg [18]. At month 6, patients with UFC levels above the ULN range could have had their doses increased by 300 μg BID (to a maximum of 1200 μg BID). Throughout the 12-month treatment period, the dose of pasireotide could be decreased in 300-μg increments BID, if required, because of adverse events such as uncontrolled hyperglycemia. Patients who had measurable pituitary tumors according to MRI assessments at baseline and at months 6 and/or 12 were evaluated in this analysis.

### UFC levels

UFC levels were assessed monthly through month 6 and then every 3 months thereafter, and the assays were performed in a central laboratory as described previously [18]. Levels were calculated by obtaining the 24-h samples, which were collected within a 2-week period at all time points. The most recently available value (on the basis of ≥ 3 collections) between months 3 and 6 was carried forward if no UFC measurement was available for month 6. Patients who did not have valid UFC measurements available as well as those who discontinued the study medication before month 3 were considered to have uncontrolled hypercortisolism. Patients were considered to have normalized UFC (full UFC responders) if their UFC level was ≤ ULN at month 6 without a prior dose increase. Patients were considered to have partial UFC response if their level was > ULN but had a ≥ 50% reduction from baseline.

### Pituitary tumor volume

Tumor volume assessments were determined with MRI scans performed at each site at baseline and at months 6 and 12 following treatment with pasireotide (or at the time of study drug discontinuation). In this subanalysis, patients who had tumor measurements within 40 days of the baseline evaluation were included. The MRI scans were sent to a central reader (Bio-Imaging Technologies, Inc), and the reading of the images was performed by two independent radiologists who were blinded to the treatment dose as well as to the treatment time points. In case of disagreement between the 2 readers in their evaluation of the tumor volume, a third radiologist reviewed the data and selected the assessment of one of the readers. If the two readers agreed, the average tumor volume was used for the analysis. Tumor volume was assessed by extrapolation, after measuring the area of the serial MRI slices and then integrating them into a 3-dimensional structure using a computer model. The intraclass correlation coefficient (ICC) was calculated to assess the reliability of tumor volume measurements from the two readers using a random-effects analysis of variance (ANOVA) model. The ICC was 0.96, suggesting high agreement between the two readers’ assessments of tumor volume. Descriptive statistics (eg, mean) of actual tumor volumes and of changes in tumor volumes were used to represent results for each dose group. To assess frequency of tumor volume reduction after treatment, stratification of baseline tumor volume was performed based on median baseline tumor volume from patients with measurable tumors at both baseline and month 6. Number and percentages of patients with tumor volume decreases of 20%, 25%, and 50% from baseline are presented for the two treatment groups.

## Results

One hundred sixty-two patients (78% female, 22% male) were enrolled in the B2305 study (Table 1) [18]. Seventy-five patients had a measurable tumor at baseline on MRI. At baseline, median pituitary tumor volume was 0.24 cm^3^ (mean volume 1.10 cm^3^; range 0.04–12.42 cm^3^) in the 600-μg (n = 36) and 0.20 cm^3^ (mean volume 0.38 cm^3^; range 0.02–2.99 cm^3^) in the 900-μg groups (n = 39). Six patients (n = 3 in each of the treatment groups) had macroadenomas (tumor volume ≥ 1 cm^3^). Fifty-three patients had measurable tumor volume at both baseline and month 6 and were included in this analysis (600-μg group, n = 25; 900-μg group, n = 28; Table 1); only 11 of these patients had not undergone pituitary surgery. Thirty-two patients were included for whom tumor volume data were available at baseline and month 12 (600-μg, n = 14; 900-μg, n = 18; Table 1). The median baseline tumor was measured as 0.20 cm^3^.

**Table 1:** Patient characteristics

Characteristic	Month 6^a^			Month 12^b^		
	Pasireotide 600 μg BID (n = 25)	Pasireotide 900 μg BID (n = 28)	Overall (n = 53)	Pasireotide 600 μg BID (n = 14)	Pasireotide 900 μg BID (n = 18)	Overall (n = 32)
Age, mean (years)	39	39	39	39	39	39
Female, n (%)	20 (80)	26 (93)	46 (87)	11 (79)	18 (100)	29 (91)
Race/Ethnic group^c^						
Caucasian, n (%)	18 (72)	19 (68)	37 (70)	9 (64)	14 (78)	23 (72)
Black, n (%)	2 (8)	1 (4)	3 (6)	1 (7)	0	1 (3)
Asian, n (%)	3 (12)	4 (14)	7 (13)	3 (21)	2 (11)	5 (16)
Other, n (%)	2 (8)	3 (11)	5 (9)	1 (7)	2 (11)	3 (9)
Missing data, n (%)	0	1 (4)	1 (2)	0	0	0
Previous treatment						
Surgery, n (%)	20 (80)	22 (79)	42 (79)	11 (79)	13 (72)	24 (75)
Medication, n (%)	8 (32)	16 (57)	24 (45)	4 (29)	11 (61)	15 (47)
Pituitary irradiation, n (%)	0	2 (7)	2 (4)	0	2 (11)	2 (6)
UFC level^d^						
Mean, nmol/24 h	887.6	837.9	861.7	1100.1	526.4	792.8
Median, nmol/24 h	731.2	491.5	537.8	807.5	416.5	501.3
Range, nmol/24 h	265.0–4564.2	195.0–6122.8	195.0–6122.8	280.8–4564.3	195.0–1757.8	195.0–4564.3

*BID* twice daily, *SC* subcutaneous, *UFC* urinary-free cortisol

^a^Evaluable patients with measurable tumor volume data at baseline and at month 6. Two patients in the 600-μg group and three patients in the 900-μg group did not have baseline UFC measurements available

^b^Evaluable patients with measurable tumor volume data at baseline and at month 12. One patient from each group did not have baseline UFC measurements available

^c^No information on race or ethnic group was reported for one patient in the 900-μg group

^d^UFC normal range upper limit of normal, 145 nmol per 24 h

**Fig. 2:**
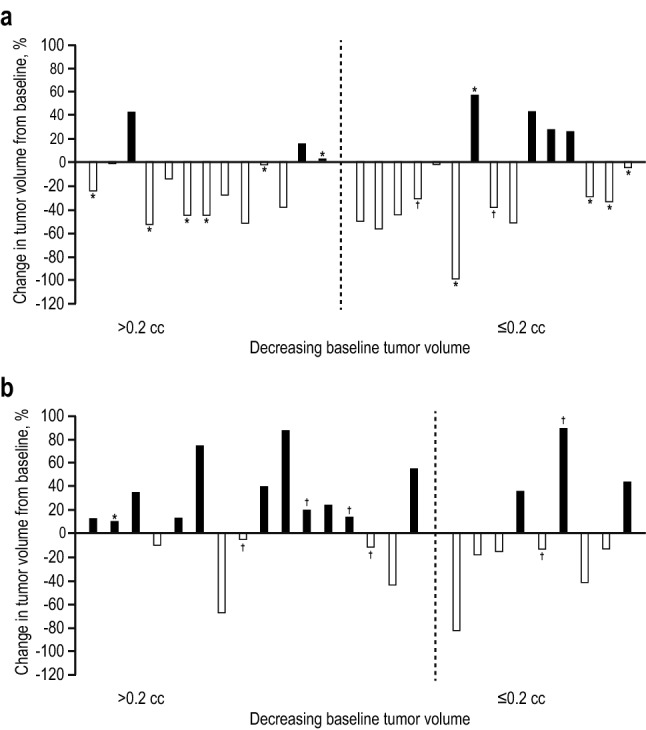
Percentage of tumor volume changes by baseline tumor volume at month 6 in **a** individual patients treated with pasireotide 900 μg and **b** individual patients treated with pasireotide 600 μg. *UFC* urinary-free cortisol. *Indicates full UFC responder. ^†^Indicates UFC partial responder

At month 6, there was a mean tumor volume decrease from baseline of 5.7% (95% confidence interval [CI] − 17.4% to 6.0%). In particular, there was a mean tumor volume increase of + 9.3% (95% CI − 8.9% to 27.5%) in the 600-μg group (n = 25) but a decrease of 19.0% (95% CI − 33.3% to − 4.7%; median − 28.9%) in the 900-μg group (n = 28). Tumor volume reduction was observed less frequently in the 600-μg group (44%) than in the 900-μg group (75%; Table 2). Tumor volume reductions of ≥ 25% were observed in 16% and 57% of patients in the 600- and 900-μg groups, respectively. Reductions of ≥ 50% of tumor volume were observed in 8% and 21% of patients in the 600- and 900-μg groups, respectively. At baseline, median pituitary tumor volumes were 0.24 and 0.20 cm^3^ in the 600- and 900-μg groups, respectively [18]. To assess frequency of tumor volume reduction from baseline at month 6 in the 53 patients with measurable tumors, patients were stratified into those with tumors > 0.2 cm^3^ and those ≤ 0.2 cm^3^ at baseline. In the 600-μg group, 5 of 16 tumors (31%) > 0.2 cm^3^ and 6 of 9 tumors (67%) ≤ 0.2 cm^3^ at baseline decreased in volume (Fig. 2). In the 900-μg group, 10 of 13 tumors (77%) > 0.2 cm^3^ at baseline and 11 of 15 tumors (73%) ≤ 0.2 cm^3^ in size at baseline decreased in volume.

**Table 2:** Changes in tumor volume at 6 and 12 months

Parameter	Month 6		Month 12	
	Pasireotide 900 μg BID (n = 28)^a^	Pasireotide 600 μg BID (n = 25)^a^	Pasireotide 900 μg BID (n = 18)^b^	Pasireotide 600 μg BID (n = 14)^b^
Total full UFC responders, n (%)	11 (39)	1 (4)	9 (50)	1 (7)
Patients with tumor volume decrease, n (%)	21 (75)	11 (44)	16 (89)	7 (50)
Full UFC responders, n (%)	9 (43)	0	8 (50)	1 (14)
Patients with tumor volume decrease ≥ 20%, n (%)	16 (57)	4 (16)	14 (78)	5 (36)
Full UFC responders, n (%)	7 (44)	0	7 (50)	1 (20)
Patients with tumor volume reduction ≥ 25%, n (%)	16 (57)	4 (16)	13 (72)	5 (36)
Full UFC responders, n (%)	7 (44)	0	7 (54)	1 (20)
Patients with tumor volume reduction ≥ 50%, n (%)	6 (21)	2 (8)	7 (39)	4 (29)
Full UFC responders, n (%)	2 (33)	0	5 (71)	1 (25)
Patients with tumor volume increase, n (%)	7 (25)	14 (56)	2 (11)	7 (50)
Full UFC responders, n (%)	2 (29)	1 (7)	1 (50)	0
Patients with tumor volume increase ≥ 20%, n (%)	5 (18)	10 (40)	2 (11)	4 (29)
Full UFC responders, n (%)	1 (20)	0	1 (50)	0

*UFC* urinary-free cortisol

^a^Evaluable patients with measurable tumor volume data at baseline and at month 6

^b^Evaluable patients with measurable tumor volume data at baseline and at month 12

Differences were observed in the amount of tumor volume reduction in the 2 groups. In the 600-μg group, average changes from baseline in tumor volume (± standard error of mean) were 15.6% ± 10.0% and − 1.8% ± 17.0% in patients with tumors > 0.2 cm^3^ and ≤ 0.2 cm^3^, respectively. Conversely, in the 900-μg group, the average change from baseline in tumor volume (± standard error of mean) was –18.8% ± 8.2% and − 19.2% ± 11.2% in patients with tumors > 0.2 and ≤ 0.2 cm^3^, respectively. In patients with macroadenomas who were assigned to the 600-ug group, tumor volume increased in all 3 patients from baseline at month 6 (by 13%, 11%, and 36%). In patients assigned to the 900-ug group at month 6, a total of 2 out of 3 patients (67%) had a decrease in tumor volume from baseline by 25% and 1%, and 1 patient had a 43% increase in tumor volume.

At month 6, results of the descriptive analysis do not sufficiently establish a relationship between change in tumor volume and achievement of UFC control in either treatment group. In the 600-μg group, 1 of 25 (4%) patients with macroadenoma at baseline was a UFC full responder, and the patient had an 11% increase in tumor volume. Six (24%) patients were partial responders (ie, UFC > ULN but ≥ 50% reduction from baseline); 3 (12%) patients exhibited < 20% reductions in tumor volume, whereas tumor volume increased in 3 (12%) patients. Among 18 (72%) UFC nonresponders, 4 (22%) experienced < 20% reductions in tumor volume and 4 (22%) patients exhibited ≥ 20% decrease in tumor volume. Ten nonresponders of 18 (56%) experienced an increase in tumor volume. In the 900-μg group, 11 of 28 patients (39%) were full UFC responders, and tumor volume was reduced in 9 of 11 (82%) full UFC responders. Two full UFC responders (7%) exhibited increased tumor volume. Seven of 11 full responders (64%) exhibited ≥ 20% reduction in tumor volume, including 1 patient with macroadenoma at baseline, and 2 (18%) exhibited < 20% reduction. Two of 28 (7%) patients were partial responders, and ≥ 20% reductions in tumor volume were observed in both patients. Tumor volume was reduced in 10 of 15 (67%) patients who were not UFC responders, and the remaining 5 patients (33%) showed an increased tumor volume. Seven of 15 nonresponders (47%) experienced ≥ 20% reductions in tumor volume, and 3 patients (20%) experienced < 20% tumor volume reductions.

**Fig. 3:**
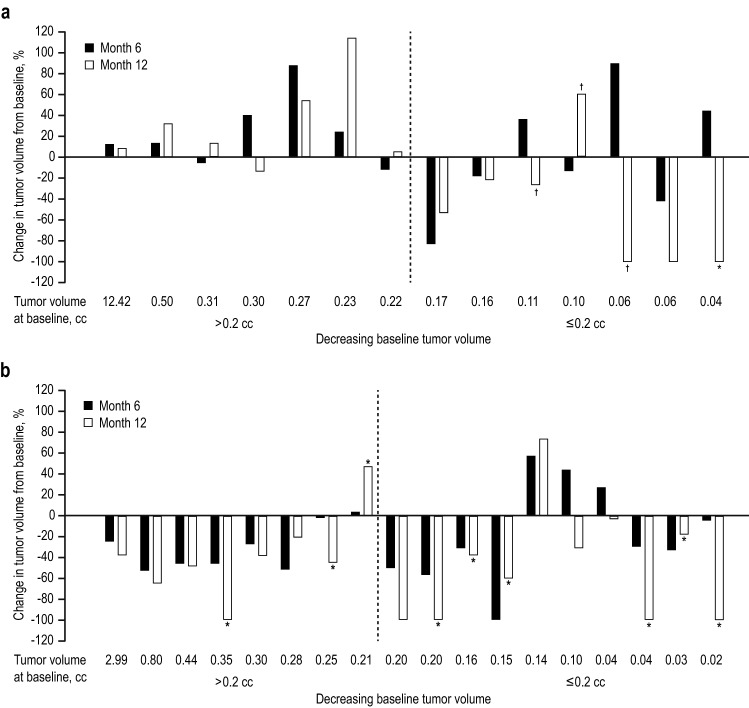
Percentage of tumor volume changes by baseline tumor volume at month 6 (black bar) and at month 12 (white bar) in **a** individual patients treated with pasireotide 600 μg and **b** individual patients treated with pasireotide 900 μg. *UFC* urinary-free cortisol. *Indicates full UFC responder. ^†^Indicates UFC partial responder

Although the number of evaluable patients was somewhat smaller at month 12 compared with month 6, a similar trend in tumor volume change was observed (Fig. 3). At month 12, there was a mean tumor volume decrease of 28.6% (95% CI − 49.6% to − 7.6%; median, 34.7%) from baseline. In the 600-μg group, there was a mean tumor volume decrease of 9.1% (95% CI 46.3% to 28.0%; median 4.2%; n = 14) and 7 of 14 (50%) patients experienced reductions in tumor volume (≥ 25% reduction in 5 patients and ≥ 50% in 4 patients). In this group, 2 of 3 patients (67%) with tumor volume reduction at month 6 experienced further reductions in tumor volume at month 12. The patient with macroadenomas and a 13% increase in tumor volume at month 6 experienced tumor volume decrease; the net increase from baseline at month 12 for this patient was 8%. One of 14 patients (7%) at month 12 achieved full control levels and 3 other patients achieved partial control of UFC levels. Seven patients (50%) with increased tumor volume at month 12 in the 600-μg group did not achieve full control of UFC levels; however, 1 of 7 patients (14%) achieved partial control of UFC levels. The remaining 6 patients (99%) continued to have uncontrolled UFC.

At month 12, in the 900-μg group (n = 18), a decrease in mean tumor volume of 43.8% (95% CI 68.4% to 19.2%; median 42.0%) was observed. Sixteen of 18 (89%) patients experienced reductions in this group (≥ 20% reduction in 14 patients, ≥ 25% in 13 patients, and ≥ 50% in 7 patients). Thirteen of 16 (81%) patients with reductions in tumor volume at month 6 experienced further reductions in tumor volume at month 12. The patient with macroadenomas with 25% tumor volume reduction at month 6 from baseline experienced further reduction at month 12, with a total tumor reduction of 38% from baseline. Eight of 16 patients with tumor volume reduction (50%) at month 12 achieved full control, and none of the other patients in this 900-μg group achieved partial control of UFC levels at month 12. One of 2 patients (50%) who had increases in tumor volume at month 12 achieved full control of UFC levels; the remaining patient had uncontrolled UFC.

Of the 52 patients whose pituitary lesions were not detectable on MRI at baseline, only 1 of 15 (6.7%) patients receiving treatment at 6 months and 2 of 37 (5.4%) patients receiving treatment for 12 months developed a tumor visible on follow-up MRI.

## Discussion

The B2305 Phase 3 trial demonstrated substantial decreases in UFC levels, improvement in clinical signs of disease, and decreases in mean pituitary tumor volume in patients with CD who were treated with pasireotide [18, 19]. Our post hoc analysis was performed to further assess the effects of pasireotide on corticotroph tumor volume among patients enrolled in the trial. The results of the current study demonstrate a measurable decrease in MRI-detectable pituitary tumor volume in a majority of patients with CD who were treated with pasireotide 900 μg (75% at month 6; 89% at month 12), but in a smaller percentage of patients treated with pasireotide 600 μg (44% at month 6; 50% at month 12).

Interestingly, no relationship was established between tumor size at baseline (when considering whether tumors were > 0.2 cm^3^ or ≤ 0.2 cm^3^) and the frequency of change in tumor volume in response to pasireotide 900 μg at month 6. Although 2 of 3 patients with macroadenomas at baseline experienced tumor reductions at month 6 with the 900-μg dose of pasireotide, the small sample size precludes any meaningful conclusions for this subgroup. In contrast, patients treated with pasireotide 600 μg with tumors ≤ 0.2 cm^3^ were more likely to experience reductions in tumor volume than those with larger tumors. Interpretations of data regarding changes in tumor volume in tumors ≤ 0.2 cm^3^ are challenging because of the difficulties in accurately assessing the size of the tumor. Therefore, the frequency of tumor volume reduction in the smallest tumors in the study may be underrepresented, and it is possible that reductions in tumor volume may occur at either of the pasireotide 600 μg or 900 μg doses. Unfortunately, the number of patients with measurable tumor volume who had not undergone pituitary surgery was too few to allow for a meaningful separate analysis.

In 52 patients with residual corticotroph tumors not visible on MRI at baseline, only 3 patients developed tumors visible on MRI within 6 or 12 months of therapy with pasireotide. Tumor progression has been reported as 39% at 3 years, plateauing to 47% at 7 years following bilateral adrenalectomy in 46 patients with persistent or recurrent CD despite no visible residual tumor on imaging prior to adrenal surgery [20]. Comparative data are lacking on the progression of corticotroph tumors over time with or without other medical interventions and without bilateral adrenalectomy for the management of CD.

Somatic mutations in the ubiquitin-specific peptidase 8 gene (*USP8*), which prevents the degradation of epidermal growth factor receptors, were recently identified in 40% to 62% of corticotroph tumors from patients with CD [21–23]. The majority of tumors with *USP8* mutations were smaller and produced relatively more ACTH than those without *USP8* mutations. Levels of SSR_5_ receptors were significantly higher in the *USP8-*mutated tumors than in the wild-type tumors, suggesting that pasireotide may be more effective in the *USP8*-mutated tumors, which are mostly microadenomas, than in the macroadenomas, which less frequently carry *USP8* mutations [21]. However, the status of *USP8* and of SSR_5_ receptors in the smallest tumors (close to 50%, not detectable by MRI) is largely unknown because it is difficult to obtain sufficient tumor tissue to characterize their molecular status.

No correlation between tumor volume reduction and UFC control has been established, thereby suggesting that pasireotide may have independent actions on pathways that regulate hormone secretion and on those that regulate tumor growth. At month 6, in the 900-μg group, 43% (9 of 21) of patients with any tumor reduction from baseline were classified as full UFC responders, and 10% (2 of 21) of patients were partial UFC responders; in the 600-μg group, none of the patients with any tumor reduction from baseline were classified as full UFC responders, and 27% (3 of 11) of patients were partial UFC responders. At month 12, in the 600-μg group, 14% (1 of 7) of patients with any tumor reduction from baseline were classified as full UFC responders, 29% (2 of 7) of patients were partial UFC responders, and at month 12, in the 900-μg group, 50% (8 of 16) of patients with any tumor reduction from baseline were classified as full UFC responders, and of the remaining patients had uncontrolled UFC. In addition, of the 9 patients who experienced increases in tumor volume at month 12, 1 (11%) patient in the 900-μg group attained full UFC control, and 1 (4%) patient in the 600-μg group was considered a partial responder. These observations preclude any substantial correlation between UFC level normalization and tumor volume reduction. However, not all patients experience tumor volume reduction, and future studies may help identify factors that are important determinants of tumor volume response.

Limitations of the current analysis include the substantial number of patients enrolled in the study (69.5% of patients in the 600-μg group and 65% of those in the 900-μg group) who did not receive pasireotide for prolonged periods of time and thus lacked data for tumor volume measurements after baseline at months 6 and 12. Additionally, the apparent imbalance in baseline mean and median UFC levels between the 2 groups may have affected the overall efficacy observed in the study [18]. However, this analysis indicates a limited relationship between pasireotide-induced change in corticotroph tumor volume and UFC control, suggesting that UFC imbalances may not have affected tumor volume change in this study. Another potential limitation was the variability in assessing tumor volume changes, specifically, the accuracy of detecting small changes in volume in smaller tumors. For this reason, a threshold of ≥ 25% was considered to be a substantial change in tumor volume, which is consistent with other studies [24–28].

Taken together, the results of the current analysis demonstrate that treatment with pasireotide, a pituitary-directed medical therapy that targets SSRs, can frequently lead to radiologically measurable reductions in pituitary tumor volume in patients with CD. Tumor volume reduction is especially relevant in patients with larger microadenomas, suggesting that pasireotide is an attractive option for these patients, especially in cases in which patients cannot undergo transsphenoidal surgery or do not respond to surgical management of disease.

## Data Availability

The datasets generated during and/or analyzed during the current study are available from the corresponding author on reasonable request.
